# Non-completion of upper secondary school among female and male young adults in an Arctic sociocultural context; the NAAHS study

**DOI:** 10.1186/s12889-016-3644-2

**Published:** 2016-09-13

**Authors:** Elisabeth Valmyr Bania, Stian Lydersen, Siv Kvernmo

**Affiliations:** 1 Department of Clinical Medicine, Faculty of Health Sciences, University of Tromsoe; The Arctic University of Norway, N-9037 Tromsoe, Norway; 2 Faculty of Medicine, Regional Centre for Child and Youth Mental Health and Child Welfare, (RKBU Central Norway), Norwegian University of Science and Technology (NTNU), N-7491 Trondheim, Norway; 3 Department of Child and Adolescent Psychiatry, Division of Child and Adolescent Health, University Hospital North Norway, N-9038 Tromsoe, Norway

**Keywords:** Non-completion of upper secondary school, Gender differences, Adolescents, Reading –and writing difficulties, Mental health, Sami, Indigenous, Religion

## Abstract

**Background:**

Education is closely associated with health. Non-completion of upper secondary school influences academic achievement, employment, income and personal well-being.

The purpose of the study is to explore predictors of non-completion of upper secondary school among female and male young adults in relation to mental health and educational factors in a socio-cultural, Arctic context.

**Methods:**

The Norwegian Arctic Adolescent Health Study (NAAHS) is a cross-sectional, school-based survey that was conducted in 2003–2005. Eighty-three percent of the population of 5,877 10th graders participated; 49.1%females, 450 reported indigenous Sami ethnicity, and 304 reported Laestadian affiliation. Data from NAAHS were merged with registry data from the National Education Database (NUDB) Norway for 3,987 adolescents who gave their consent for follow-up studies.

**Results:**

Non-completion of upper secondary school was 36.9 % among females and 36.6 % among males. Among females, predictors for non-completion were related to mental health symptoms, and among males, to residency in the northernmost and remote areas and self-reported functional difficulties at school, home and in leisure activities due to mental health problems.

There was marginal significance between ethnicity and non-completion of upper secondary school, measured at 41.3 % for Sami and 36.8 % for non-Sami, respectively.

**Conclusions:**

The gender differences found in this study emphasize the need for gender-specific interventions in preventing non-completion of upper secondary school. There is a need to recognize and treat extensive pro-social behaviour and social problems in young females. Young males from remote areas and those who in early adolescence struggle with functional impairment due to mental health problems need early interventions in lower secondary school. Enhancing parents’ and teachers’ ability to detect symptoms and problems as well as low-threshold health services starting in primary school can be effective means. Education, mental health and social inclusion are prominent factors for future employment, income and independent living for young people. Future research should focus more on gender-specific patterns of risk and protective factors for completion of upper secondary school.

## Background

Non-completion of upper secondary school is a widespread phenomenon and a public health issue as education has a strong impact on living conditions, personal well-being, and health [1]. Research into the underlying mechanisms of this phenomenon has shown various results for different adolescent groups, complicating general preventive initiatives [2, 3] Social, educational, behavioural, and mental health factors are all found to contribute, but to differing extents [1, 4–10]. Our aim in this study is to examine how various aspects of young adolescence such as social and cultural environments, mental health, and educational characteristics predict later non-completion of upper secondary school among females and males in an Arctic sociocultural context.

Mental health issues and behaviours representing a risk to health are found to influence performance in upper secondary school [11] and completion [12, 13]. Youngsters diagnosed with ADHD (Attention Deficit Hyperactivity Disorder) are more likely to not complete upper secondary school due to attention problems associated with ADHD and comorbid learning disabilities [14]. Breslau et al. [14] suggested that internalizing symptoms such as depression does not influence youngsters’ completion of upper secondary school, while other studies show a significant association between early adolescents depressive symptoms and later non-completion of upper secondary school [3, 12]. In studies of younger adolescents, deviant behaviour such as poor conduct are contributing factors to non-completion of upper secondary school by obstructing educational achievements [3, 9].

Newcomb, Abbott [15] found that structural strain such as gender, ethnicity and socio-economic status (SES) along with academic achievement reflected in marks influenced completion of upper secondary school. Based on current research, it is estimated that an average of 17 % of all young people, mainly males, within the Organization for Economic Co-operation and Development (OECD) countries will not complete upper secondary school over their lifetimes [16, 17]. Young women are now more likely than young men to complete upper secondary school in almost all OECD countries [16, 17]. The gender gap on all academic levels are widely recognized [18–20]. Female students perform better academically than male students [21], and more often complete their studies despite feeling distressed [20]. Attributes such as independence and competitiveness correspond to the stereotypical perceptions of masculinity, while interpersonal skills and cooperation abilities are more typically associated with femininity. Then again, other typically masculine traits, such as self-confidence, predict achievement and deviant behavior drop –out [13] However, completion and non-completion of upper secondary school among females and males is more complicated when multivariate factors are included. Gender-specific analysis will be helpful in conducting what Newcomb et al. [15] present as a more process-oriented investigation of non-completion of upper secondary school. Parental SES is shown to have great impact on completion and non-completion of upper secondary school, where low parental SES is associated with non-completion, and higher parental SES is associated with completion [2, 22].

Educational characteristics, such as educational aspirations, average marks and reading and writing difficulties mutually influence each other [6]. The single most prominent factor of educational aspirations on higher level is higher average marks, and for typically vocational education, lower average marks [8]. Reading difficulties do not indicate solely learning difficulties, but a lack of comprehension can impact average marks and completion or non-completion of upper secondary school [23]. Writing difficulties are often combined with reading comprehension difficulties, indicating complex difficulties and leading to poor marks and non-completion. [6, 23]. Educational skills, parental SES, the social context and mental health all influence educational aspirations [8], underscoring the importance of adolescents’ motivation to complete upper secondary school [2, 4, 24, 25] and may act as mediators or predictors of both completion and non-completion [26].

Studies outside the Arctic have addressed drop out from upper secondary school, also among indigenous people [22, 27–29]. Nevertheless, few studies in the Arctic have examined how educational factors predict non-completion of upper secondary school when controlling for the presence of mental health, educational -and sociocultural factors.

Arctic Norway, characterized by its scattered, remote and multi-ethnic population, including the indigenous Sami with their own language and culture, is overrepresented in terms of non-completion of upper secondary school when compared with OECD countries and the rest of Norway [16, 17, 30]. The non-completion rates vary from 23 to 29 %, increasing with a higher latitude and more remoteness [31]. The indigenous Sami are strongly affiliated to the Laestadian religious movement traditionally considered a Sami version of Lutheran Christianity, and is still strongly maintaining abstinence norms, conservative moral ethics and an ideal of strong family cohesion [32–35]. The sociocultural and geographical context of Arctic Norway constitutes a unique structural frame, compared to more centralized locations, with its indigenous Sami, the Lutheran Christian Laestadians and the geographical periphery. Despite these differences, the school system, as in the rest of the Norway, continues to be based on urban and mainstream values and practice [36].

Previous studies have revealed that indigenous peoples have higher non-completion rates than the majority population [37–39]. Among upper secondary school students in Sami areas in Norway in the mid-nineties, the non-completion rate was 54 % irrespective of the pupil’s ethnicity, and even higher nearly a decade later, with an alarmingly high non-completion rate of 58 % of all pupils and 63 % for males [40]. Indigenous peoples, the Sami minority included, were subjected to boarding schools, a forced change of language and loss of group culture until the late 1900s. These circumstances may have resulted in a resistance towards the school system that may have been transferred to their descendants [41, 42].

The first aim of this study was to estimate the prevalence of non-completion of upper secondary school between females and males, Sami and non-Sami in Arctic Norway. The second aim was to determine the predictive value of adolescent sociocultural factors such as residency, parental socioeconomic status, and Laestadian religious affiliation on completion of upper secondary school. Thirdly, we wanted to examine the impact of personal traits such as young people’s mental health (symptoms and functional impairment), and educational characteristics on later non-completion of upper secondary school when controlled for sociocultural factors. Finally, we wanted to examine for possible gender differences in predictors of completion and non-completion of upper secondary school.

We expected that lower parental SES, male gender, Sami ethnicity, reading and writing difficulties and mental health problems would increase the risk of non-completion of upper secondary school, whereas good educational skills, Laestadian religious affiliation and higher educational aspirations would decrease the risk.

## Methods

### Sample and procedure

The participants in the Norwegian Arctic Adolescent Health Study (NAAHS) included 4,881 of 5,877 (RR 83 %) 15/16-year-old 10th grade pupils in lower secondary schools from January 2003 to January 2005. All 10th graders in the three northernmost Norwegian counties were invited to participate.

The questionnaires were administered in a two-hour period in a classroom setting monitored by project staff, and pupils who were absent completed the survey later. The questionnaire was available in two languages: Sami and Norwegian.

The adolescents had to provide written consent for later follow-up studies including linkage to registry data. A total of 3,987 (68 %) of the invited sample gave their consent. Data on upper secondary school were missing for six students, and these were excluded from the study, leaving a working sample of 3,981 students.

In order to investigate completion and non-completion of upper secondary school, data from NAAHS were merged with registry data from the National Education Database (NUDB) from the period 2003–2013. NUDB is a national registry database, with data covering all aspects related to education (primary school, lower secondary school, upper secondary school and higher education). Upper secondary school is a universal right for all youth under the age of 25 in Norway, and is generally completed in three years. The Regional Committee for Medical Research Ethics approved the study.

### Measures

#### Outcome variable from NUDB

*Completion of upper secondary school* is defined as achieving a complete diploma of upper secondary school within five years after completing secondary school. Not having completed upper secondary school within five years after lower secondary school was defined as “non-completion”. Completion of upper secondary school was used as the reference group criterion.

#### Explanatory variables from the NAAHS study

*Gender*. Female gender was used as the reference group.

*Residency* refers to the county where the adolescent lived during lower secondary school. The three northernmost counties in Norway were compared: Nordland, Troms and Finnmark of which Finnmark is the northernmost and most remote and most sparsely populated county. Nordland county, the southernmost, has the greatest number of inhabitants and is used as the reference group.

*Sami ethnicity* was measured by an assessment of parents’ ethnicity, Sami language competence in parents, grandparents and the participants, and ethnic self-identification. Participants who had one or more of these affiliations present were classified as having Sami ethnicity [43]. Non-Sami ethnicity was used as the reference group.

*Socioeconomic status (SES):* Information was obtained about the parents’ occupation and classified according to the International Standard Classification of Occupation (ISCO-88) [44], and later reclassified into five categories, here labelled Parental SES. The categories used were: “Higher administrative position” [1], “Intermediate position” [2]; “Lower administrative position” [3]; “Primary industry” [4] and “Blue-collar worker” [5].” Unknown position”, reported by only a few participants, and recoded into the missing group. Option [1] was used as the reference group.

*Laestadian affiliation* was measured by the youth’s self-reports of their own, or the parents’ or the grandparents’ affiliation to the Laestadian religious movement. Participants having one or more of the affiliations were classified as having Laestadian affiliation. Non-Laestadian affiliation was used as the reference group.

#### Mental health

*Strength and Difficulties Questionnaire* (SDQ) [45] consists of five subscales. We used all subscales as measures of psychosocial health: the Emotional Symptom scale (SDQ-emotions; Cronbach’s alpha = .70); the Pro-Social Behaviour scale (SDQ-pro-social; Cronbach’s alpha = .65) and the Peer Problem Scale (SDQ-peer; Cronbach’s alpha = .52); the Hyperactivity-Inattention scale (SDQ-hyper; Cronbach’s alpha = .64); the Conduct Problem scale (SDQ-conduct; Cronbach’s alpha = .47). The subscales have five items each with scores from 0 to 2 on each item, indicating; 0 = Not correct, 1 = Correct sometimes 2 = Totally correct. The Pro-Social subscale has inverse scores. The total score for these subscales ranged from 0 to 10, with the lowest score indicating the least amount of difficulty, and for pro-social behavior the highest score (up to 10) indicating the least amount of difficulty. Each question was scored from 0 to 2, with 0 indicating no problems and 2 indicating great worries and large problems. The scales were operationalized on the basis of the mean scores of the five questions.

The subscale SDQ-Impact scale (SDQ-impact, Cronbach’s alpha = 0.69) was used, where a score of 10 implies the greatest functional impairment due to mental health problems in home life, friendships, classroom activities and leisure activities. The scale consisted of 5 questions, and operationalized by mean score.

#### Educational characteristics

*Average marks are* based on the four major subjects: mathematics, Norwegian, English and social sciences in lower secondary school. The Norwegian system of school marks ranges from 1 to 6 (1–2 = poor, 3 = average, 4 = good, 5 = very good and 6 = excellent). For this variable to be included in the analyses, a reported mark in at least three out of the four subjects must be present.

*Reading –and writing difficulties (RWD)* is measured by the question “Have professionals stated that you have or have had reading –and writing difficulties?” with the following options; “Yes, large problems”, “Yes, medium problems” and “Yes, some problems”, or “No problems”. Positive score on one of the first three options was scored as *“yes”* [1]. Not reporting RWD was used as the reference group.

*Educational aspirations* were measured by the question “What is the highest educational level you plan to reach?” from the cross-sectional survey NAAHS, completed 2003–2005. The students could only respond to one option;

“University or junior college on high level” (teacher holding a master’s degree, solicitor, civil engineer, dentist, doctor, psychologist, master in business administration, etc.) [1], “University or junior college at middle level” (Norwegian university degree (3.5– 4.5 years), teacher, social worker, nurse, police, engineer, journalist, etc.) [2], “Diploma upper secondary school” [3], “Vocational education at upper secondary school level (chef, hairdresser, builder, electrician, assistant in health and social care etc.) [4], “One year in upper secondary school” [5], “Other: blank to be filled in” [6], “I have not decided” [7].

The options were recoded into 4 categories: higher [1], intermediate [2], lower [3], and undecided [4] (undetermined on the choice of profession).

The option ‘undecided’ was used as the reference group.

### Statistical analyses

Groups were compared using Pearson’s chi squared test for categorical data, and Student’s *t*-test for continuous data. Logistic regression analyses were carried out with completion and non-completion of upper secondary school as the dependent variable, unadjusted and fully adjusted separately for females and males. In the logistic regression analyses, missing values were handled using multiple imputations (MI). All variables used in the subsequent analyses were included in the imputation model. One hundred data sets were imputed, as recommended by van Buuren [46]. The MI analyses gave substantially the same results as complete case analyses (CC). Only results from MI analyses are reported.

Two-sided *P*-values ≤0.05 were considered statistically significant. SPSS 22 was used for all analyses.

## Results

A total of 10.0 % of the sample were indigenous Sami. More Sami youth reported Laestadian affiliation and primary industry vocations, such as reindeer herding. Regarding parental SES, significantly more Sami parents worked in the primary industry. The numbers were reversed for parental lower administrative position. The proportion of Sami students was highest in the northernmost county (Table 1).

**Table 1:** Characteristics of the sample by registry data (NUDB), based on NAAHS variables (10^th^ grade)

		Gender *N* = 3987						Ethnicity *N* = 3987				
		Females		Males		*p*-value		Sami		Non-Sami		*p*-value
	Total N (MV^a^)	N	%	N	%		Total N (MV^a^)	N	%	N	%	
Upper secondary school	3981 (6)					^ns^	3639 (348)					^§^
Completion	2518	1253	63.1	1265	63.4		2283	213	58.7	2070	63.2	
Non-completion	1463	734	36.9	729	36.6		1356	150	41.3	1206	36.8	
Ethnicity/Gender	3645 (342)					^ns^						
Sámi	365	187	10.2	178	9.8							
Non-Sámi	3280	1646	89.8	1634	90.2							
Religious group	3987					***	3645 (342)					***
Laestadian	254	100	5.0	154	7.7		253	75	20.5	178	5.4	
Non-Laestadian	3733	1882	95.0	1842	92.3		3392	290	79.5	3102	94.6	
Residency	3987					^ns^	3645 (342)					***
Nordland county	2104	1033	51.9	1071	53.6		1923	89	24.4	1834	56.0	
Troms County	1310	674	33.9	636	31.9		1195	119	32.6	1076	32.8	
Finnmark county	573	284	14.2	289	14.5		527	157	43.0	370	11.2	
Parental socio-economic status	3413 (574)					^ns^	3146 (841)					*
Higher administrative position	461	236	13.5	225	13.5		430	45	14.5	385	13.6	
Mediate administrative position	1374	684	39.2	690	41.4		1274	122	39.4	1152	40.6	
Lower administrative position	965	521	29.8	444	26.7		881	74	23.9	807	28.5	
Primary industry	168	81	4.6	87	5.2		153	25	8.1	128	4.5	
Blue collar work	445	225	12.9	220	13.2		408	44	14.2	364	12.8	
Educational characteristics (RWD)	3933 (54)					^ns^	3598 (389)					^ns^
Reading and writing difficulties	797	404	20.6	393	19.9		726	71	19.7	655	20.2	
Non-reading and writing difficulties	3136	1558	79.4	1578	80.1		2872	290	80.3	2582	79.8	
Educational aspirations	3928 (59)					***	3605 (382)					^§^
Educational aspiration higher level	865	472	24.0	393	20.1		812	86	24.0	726	22.4	
Educational aspiration intermediate level	722	453	23.0	269	13.7		664	59	16.4	605	18.6	
Educational aspiration lower level	1224	494	25.1	730	37.2		1102	95	26.5	1007	31.0	
Educational aspiration undeceive	1117	549	27.9	568	29.0		1027	119	33.1	908	28.0	

*ns* not significant

^§^ *p* ≤ 0.10 * *p* ≤ .05 ** *p* ≤ .01 *** *p* ≤ .001

^a^ *MV* missing values

Non-completion of upper secondary school was fairly equally distributed between genders, with prevalence of 36.9 % for females and 36.6 %, for males. Sami young people showed marginally significance towards non-completion (41.3 %) compared to non-Sami counterparts (36.8 %) (*p* = .05). More Sami adolescents and more males reported Laestadian affiliation compared to non-Sami and females. Females reported more academic aspirations at higher and intermediate level than males, whereas males reported more vocational (lower) aspirations than females. No significant gender difference occurred for indecisive aspirations, nor for educational aspirations between Sami and non-Sami adolescents.

The analyses revealed that for SDQ-subscales and Average marks only male gender and SDQ-impact showed statistical significance (F = 4.89, *p* = .03). (Data not shown).

There was a significantly higher rate of non-completion of upper secondary school among males residing in Finnmark County (*p* = .02) (Table 2). Other demographic characteristics, such as Laestadian affiliation, parental socioeconomic status, educational aspirations or reading or writing difficulties showed no significant impact on completion or non-completion in this study. Statistically significant differences were only found in the effects between females and males. To ascertain gender specific predictors of upper secondary completion and non-completion, further analyses were carried out separately for females and males.

**Table 2:** Demographic characteristics by upper secondary school completion and gender

		Upper secondary school completion *N* = 3987									
		Female					Male				
		Completion			Non-completion		Completion			Non-completion	
	Total N (MV^a^)	N	%	N	%	*p*-value	N	%	N	%	*p*-value
Upper secondary school	3981 (6)	1253	63.1	734	36.9		1265	63.4	729	36.6	
Ethnicity/Gender	3639 (348)					^ns^					^§^
Sami	363	109	58.9	76	41.1		104	58.4	74	41.6	
Non-Sami	3276	1034	62.9	610	37.1		1036	63.5	596	36.5	
Religious group	3978 (9)					^§^					^ns^
Laestadian	253	55	55.6	44	44.4		97	63.0	57	37.0	
Non-Laestadian	3725	1195	63.5	690	36.5		1168	63.5	672	36.5	
Residency	3981 (6)					^ns^					*
Nordland county	2103	664	64.3	369	35.7		694	64.9	376	35.1	
Troms County	1307	420	62.6	251	37.4		410	64.5	226	35.5	
Finnmark county	571	169	59.7	114	40.3		161	55.9	127	44.1	
Parental socio-economic status	3413 (574)					^ns^					^ns^
Higher administrative position	461	143	60.6	93	39.4		147	65.3	78	34.7	
Mediate administrative position	1374	448	65.5	236	34.5		438	63.5	252	36.5	
Lower administrative position	965	327	62.8	194	37.2		287	64.6	157	35.4	
Primary industry	168	49	60.5	32	39.5		47	54.0	40	46.0	
Blue collar work	445	143	63.6	82	36.4		139	63.2	81	36.8	
Educational characteristics	3927 (60)					^§^					^ns^
Reading and writing difficulties (RWD)	794	266	66.3	135	33.7		241	61.3	152	38.7	
Non-reading and writing difficulties	3133	967	62.1	590	37.9		1008	64.0	568	36.0	
Educational aspirations	3928 (59)					^ns^					^ns^
Educational aspiration higher level	865	309	65.5	163	34.5		266	67.7	127	32.3	
Educational aspiration intermediate level	722	269	59.4	184	40.6		173	64.3	96	35.7	
Educational aspiration lower level	1224	314	63.6	180	36.4		453	62.1	277	37.9	
Educational aspiration undeceive	1117	349	63.6	200	36.4		351	61.8	217	38.2	

*ns* not significant

^§^ *p* ≤ 0.10 *p ≤ .05 **p ≤ .01 *** *p* ≤ .001

^a^ *MV* missing value

The gender-specific analyses for males showed that residency in Finnmark county predicted a higher rate of non-completion of upper secondary school, both in unadjusted and fully adjusted analyses (OR:1.46, *p* = .01, OR:1.42, *p* = .02) (Table 3). Functional impairment among males due to mental health problems in lower secondary school (SDQ-impact) predicted upper secondary non-completion in both unadjusted and fully adjusted analyses (OR:1.11, *p* = .03, OR:1.11, *p* = .04) (Table 3). Lack of educational aspirations at higher level among males showed to have a marginal significant association of non-completion of upper secondary school (OR:0.77, *p* = .06, OR:0.75, *p* = .06) in the unadjusted and fully adjusted analyses.

**Table 3:** Logistic regression analysis with non-completion of upper secondary school as dependent variable, by gender. Unadjusted (only one covariate), and fully adjusted with all the listed covariates included. Results (OR, 95 % CI) from MI analyses

Non-completion of upper secondary school				
Covariate	Female		Male	
	Unadjusted	Fully adjusted	Unadjusted	Fully adjusted
Socio demographic factors				
Sámi ethnicity	1.21(0.89–1.65)	1.09(0.78–1.53)	1.25(0.91–1.72)	1.08(0.76–1.54)
Laestadian affiliation	1.39(0.92–2.09)	1.36(0.89–2.08)	1.02(0.73–1.44)	0.99(0.69–1.42)
Troms County^a^	1.08(0.88–1.32)	1.09(0.89–1.34)	1.02(0.83–1.25)	1.04(0.84–1.29)
Finnmark county^a^	1.21(0.93–1.59)	1.183(0.89–1.58)	1.46(1.12–1.90)**	1.42(1.06–1.90)*
Parental SES medium administrative position^b^	0.81(0.60–1.11)	0.80(0.59–1.09)	1.09(0.79–1.49)	1.04(0.75–1.43)
Parental SES lower administrative position^b^	0.921(0.67–1.26)	0.903(0.65–1.25)	1.03(0.74–1.44)	0.93(0.65–1.32)
Parental SES primary industry^b^	1.05(0.63–1.74)	1.00(0.59–1.69)	1.61(0.98–2.66)	1.36(0.80–2.28)
Parental SES blue–collar worker^b^	0.89(0.61–1.31)	0.86(0.58–1.28)	1.07(0.73–1.56)	0.94(0.63–1.41)
Adolescent mental health factors				
SDQ-peers	1.05(0.99–1.11)	1.07(1.01–1.14)*	1.01(0.98–1.07)	0.99(0.93–1.06)
SDQ-emotions	0.99(0.95–1.03)	0.95(0.91–1.00)*	1.02(0.97–1.07)	1.01(0.94–1.07)
SDQ-hyperactivity	1.02(0.98–1.06)	1.03(0.98–1.09)	1.02(0.97–1.06)	0.98(0.93–1.04)
SDQ-conduct	1.01(0.95–1.08)	0.99(0.93–1.08)	1.03(0.98–1.09)	1.01(0.94–1.08)
SDQ-prosocial	1.05(0.99–1.11)	1.08(1.01–1.15)*	0.97(0.93–1.02)	0.98(0.93–1.04)
SDQ-impact	1.03(0.97–1.10)	1.04(0.97–1.12)	1.11(1.01–1.21)*	1.11(1.01–1.23)*
Educational factors				
Reading and writing difficulties (RWD)	0.83(0.66–1.05)	0.86(0.68–1.08)	1.12(0.89–1.40)	1.14(0.90–1.44)
Average mark	0.96(0.85–1.09)	0.99(0.85–1.17)	0.90(0.80–1.02)	0.94(0.81–1.10
Educational aspirations				
Educational aspiration lower level^c^	1.00(0.78–1.29)	0.97(0.74–1.26)	0.99(0.79–1.24)	1.01(0.79–1.28)
Educational aspiration intermediate level^c^	1.19(0.92–1.54)	1.20(0.93–1.56)	0.90(0.66–1.21)	0.93(0.68–1.27)
Educational aspiration higher level^c^	0.92(0.71–1.19)	0.93(0.71–1.22)	0.77(0.59–1.01^)§^	0.75(0.56–1.01)^§^

*SDQ* Strengths and Difficulties Questionnaire, *RWD* Reading–and writing difficulties

^§^ *p* ≤ 0.10 * *p* ≤ .05 ** *p* ≤ .01 *** *p* ≤ .001

Reference groups:

^a^Nordland county

^b^Parental SES- Higher administrative position

^c^Educational aspirations undecided

For females in the fully adjusted analyses, several mental health symptoms such as peer problems (OR:1.07, *p* = .04), extensive pro-social behaviour (OR:1.08, *p* = .03) and less emotional problems (OR:0.95, *p* = .05) predicted non-completion of upper secondary school.

The impact of parental SES, ethnicity, reading and writing difficulties, Laestadian affiliation and average marks were not significant for non-completion of upper secondary school of any gender in the analyses.

## Discussion

To the best of our knowledge, this is the first study to explore the prevalence and the prediction of completion and non-completion of upper secondary school among females and males in a representative cohort of indigenous and non-indigenous young people in a multi-ethnic Arctic area. To achieve this, a broad variety of factors in early adolescence such as ethnic, social, religious and geographical context, mental health and educational characteristics were linked with later upper secondary school outcome from a registry database.

The rate of non-completion of upper secondary school rate in Arctic Norway was high, and higher than in the rest of Norway [31] and other OECD countries [16, 17]. Although marginal significance emerged in the unadjusted analyses, there were no ethnic differences in non-completion of upper secondary school between Sami and non-Sami adolescents when controlling for other variables. The finding is surprising compared with non-completion rates among other indigenous peoples in the Arctic and elsewhere and from previous reports from Sami areas [37, 39, 40, 47]. The unexpected finding can be understood as an indication of higher equality and equity between the Sami and non-Sami population regarding education and socioeconomic status than for other indigenous groups in the Arctic [39, 40, 48]. Inequity affecting non-completion of upper secondary school in this study seems to be more associated with the regional context affecting all ethnic groups equally than it is associated with ethnicity.

In contrast to our hypotheses and findings from other studies, no gender difference occurred in prevalence [16, 17, 40]. However, the present study showed different patterns of non-completion predictors between males and females, and these need to be taken into consideration [3, 15]. The most prominent finding among males in this registry study is the impact of residency on non-completion of upper secondary school in the northernmost and most sparsely populated county, Finnmark. Rural students and their educational performance is an important topic in educational research worldwide [36, 47, 49, 50]. The term opportunity structure, developed by Cloward and Ohlin [51] is a key concept in understanding that different contexts constitute different opportunity structures. Examples of such opportunities, or lack of such, are geographical closeness to educational institutions and range of study programmes, as well as access to labour markets. Knowledge of the local opportunity structure must be known and acknowledged by individuals to be able to understand and explain empirical findings in a specific context [36]. Green and Corbett [49] found that place of residency is important in educational attainment and influences completion and non-completion of upper secondary school, where remoteness can contribute towards a lower completion rate. The place of residency is relevant in sparsely populated Arctic Norway. Great geographical distances, small communities and the periodic lack of, or high turnover, of trained teachers are more prevalent factors. School facilities at the upper secondary level are lacking in many municipalities, particularly in Finnmark county. Students therefore have to commute long distances to their schools and must live away from their families. This living condition entails limited parental support and involvement and less social control. Deviant peer support or health risk behaviour such as substance abuse can also be a risk when there is less social control and may contribute towards lowering the adolescent’s motivation [8, 13, 15, 18]. All these factors involving a lack of support and control can contribute to non-completion of upper secondary school. Smaller schools are noted for a limited number of study programme options and smaller classes with few peers. In addition, the anxiety that various study programmes or programme durations may be curtailed can also contribute to an atmosphere of uncertainty for the youngsters and may be a factor in lowering motivation to complete educational programmes [52]. The geographical context and the rough climate can make it less attractive for the most qualified teachers to apply for work in the remote Arctic north. These are factors that may indirectly influence the students through less support, reduced involvement and limited motivation from the teachers, and thus lead to non-completion. The reasons why this occurred for males, and not for females, are not clear. One explanation may lie in the fact that males develop and mature later in adolescence [53, 54] and are therefore in greater need of parental support and role models [41, 42]; another is the fact that young males frequently have access to employment and income in a primary industry, either reindeer herding or the fishing industry, through family entitlements or community networks. The theoretical understanding of the gender-specific phenomenon has been thoroughly explained in other studies. This includes the terms presented by Kao & Tiendas [55]: blocked opportunity, oppositional identity and status attainment, referring to lack of, or lacking access to social mobility, or rejection of rational choices in understanding educational aspirations of minority youths [8]. Some of these mechanisms may be relevant for understanding upper secondary school outcome among young adolescents in this study, not in an ethnic but in a gender perspective.

The second finding among males in this study showed that functional impairment due to mental health strains is associated with non-completion, rather than the level of mental health symptoms as shown among females. Maintaining everyday life with friends, family and school and reducing stress seem to be crucial for well-being and coping for males, while reducing symptoms of mental health problems are more crucial for females.

Studies have revealed that higher parental SES and higher average marks are associated with a higher level of educational aspiration, including among males [8]. This study has shown marginal significance when controlling for other variables, higher-level educational aspirations hinder non-completion of upper secondary school among males.

The present study revealed that non-completion of upper secondary school in females was associated with various mental health problems in lower secondary school, supported by studies showing that females with emotional symptoms are less likely of not completing upper secondary school. [7, 14]. Previous studies of educational aspirations revealed that negative peer support is a significant factor in both higher and lower educational aspirations [8], and a predictor of lower educational attainment [13] but also work exclusion [56, 57]. Females with few or no friends may more easily drop out of school because they lack a social network [58]. Lack of social cohesion in a larger social network among females is also a predictor of depression and thereby non-completion [3]. The personal awareness of the adolescent must be related not only to the number of friends [59] but also the social cohesion, frequency and ties within the social network influencing the friendships [58]. Emotional well-being and friendship are shown to be associated, but emotional symptoms also occur irrespective of social cohesion [12].

The gender-specific findings among females that peer problems and more pro-social behaviour are significant for non-completion of upper secondary school should encourage more focus on young females with social problems and extensive social engagement. The social problems and social engagement can reduce their potential to complete school [9, 10] and later pursue employment opportunities, personal well-being and better health [1]. Females with emotional problems, such as anxiety and depression, are troubled mentally, and still manage to stay in school and complete upper secondary school [7, 14].

Laestadian affiliation with its abstinence norms and conservative moral ethics did not increase the rate of completion of upper secondary school. Our finding may reflect a younger adolescent population with fewer harmful behaviour patterns that are identified as risk factors in other studies, such as smoking [10], substance use [7, 14], school absenteeism [10], along with early sexual activity and teenage pregnancy [1].

In contrast to previous studies, we found no significant association between non-completion and reading and writing difficulties in lower secondary school [10] or average marks [15], showing that these factors did not represent a challenge for non-completion of upper secondary school among Arctic young people.

When one adjusts for potential confounders in a regression analysis, the effect usually decreases. For some of the analyses in Table 3, notably adolescent mental health factors for females, the effects increase somewhat in the fully adjusted model. This phenomenon can occur in observational studies, and is plausibly caused by one or more covariates acting partly as suppressors [60]. The widths of the confidence intervals remain about the same in the unadjusted and adjusted analyses, and the sample size is large compared to the number of covariates, so there are no indications of mathematical instability in the analyses.

### Strengths and limitations

The main strength of this study is the linkage of an unselected population-based study to registry data on education development and result, which makes it possible to study predictors of education completion in upper secondary school. The cross-sectional NAAHS study had a high participation rate, equal gender distribution and a representative sample of indigenous Sami.

Reliability and validity of brief scales, as SDQ, may be questioned [61, 62]. Cronbach’s alpha was applied as a measure of internal consistency reliability, with a value of .70 or more considered reliable. The Peer Problem Scale (SDQ-peer) had lower value, while subscales such as the Emotional Symptoms Scale (SDQ-emotions), the Pro-Social Behavior Scale (SDQ-pro-social), and the SDQ-Impact Scale (SDQ-impact) could be considered reliable. The validity of SDQ-peer as a psychometric tool is also questioned for certain ethnic groups of children by the lack of sociocultural sensitivity. Williamson et al. [63] exemplifies the poor fit by the lack of questions of connection to extended family, ethnic identity as well as the impact and experience of racism.

Parental socio-economic status was measured by occupation, which can be an unclear predictor. The strength is that occupations can reflect the income level to some degree, but the negative counter side is lacking knowledge of educational level.

The NAAHS data were collected 4–6 years before the outcome data in NUDB, which limited the possibility of examining the development of the predictors in late lower secondary school and during upper secondary school. Future surveys should use a longitudinal follow-up design to examine the development of predictors during upper secondary school.

The NAAHS survey was conducted during school hours, and in a classroom setting. The physical setting may have affected the response due to selection bias.

The National Education Data base (NUDB) is a high quality national registry database for education, which includes information and outcomes pertaining to all aspects of education, from primary school to higher education. The outcome variable is therefore considered reliable.

## Conclusion

Arctic Norway has equal or even greater challenges with non-completion of upper secondary school as other capitalized areas. Surprisingly, most traditional explanatory factors did not predict non-completion in this study.

Female predictors for non-completion of upper secondary school were related to social dysfunction and emotional problems, but with opposite impact. As more peer problems and extensive pro-social behavior were risk factors for non-completion, female non-completion was reduced by emotional symptoms.

Males in the remote areas were more likely not to complete upper secondary school, as were the males having problems functioning in daily life due to mental health problems.

The gender differences found in this study emphasize the need for gender-specific interventions in preventing non-completion of upper secondary school. There is a need to recognize and treat social problems in young females. Enhancing parents’ and teachers’ ability to detect symptoms and problems, along with provision of low-threshold health services, starting already in primary school, can be effective means. Young males from remote areas and those who struggle in early adolescence with functional impairment due to mental health problems need early interventions in lower secondary school. Education, mental health and social inclusion are prominent factors for future employment, income and independent living for young people. Future research should focus more on gender-specific patterns of risk and protective factors.

## Abbreviations

ADHD: Attention deficit hyperactivity disorder
CC: Complete case
MI: Multiple imputations
MV: Missing values
NAAHS: The Norwegian Arctic Adolescent Health Study
NUDB: National Education Database
OECD: Organization for Economic Co-operation and Development
RWD: Reading –and writing difficulties
SDQ: Strength and difficulties questionnaire
SES: Socio-economic status
